# Nutrient supply and accessibility in plants: effect of protein and carbohydrates on Australian plague locust (*Chortoicetes terminifera*) preference and performance

**DOI:** 10.3389/finsc.2023.1110518

**Published:** 2023-07-13

**Authors:** Jonah Brosemann, Rick Overson, Arianne J. Cease, Sydney Millerwise, Marion Le Gall

**Affiliations:** ^1^ School of Sustainability, Arizona State University, Tempe, AZ, United States; ^2^ School of Life Sciences, Arizona State University, Tempe, AZ, United States

**Keywords:** feeding behavior, locust, plant-insect interactions, nitrogen, protein, wheat

## Abstract

In contrast to predictions from nitrogen limitation theory, recent studies have shown that herbivorous migratory insects tend to be carbohydrate (not protein) limited, likely due to increased energy demands, leading them to preferentially feed on high carbohydrate plants. However, additional factors such as mechanical and chemical defenses can also influence host plant choice and nutrient accessibility. In this study, we investigated the effects of plant protein and carbohydrate availability on plant selection and performance for a migratory generalist herbivore, the Australian plague locust, *Chortoicetes terminifera.* We manipulated the protein and carbohydrate content of seedling wheat (*Triticum aestivum L.*) by increasing the protein:carbohydrate ratio using nitrogen (N) fertilizer, and manipulated the physical structure of the plants by grinding and breaking down cell walls after drying the plants. Using a full factorial design, we ran both choice and no-choice experiments to measure preference and performance. We confirmed locust preference for plants with a lower protein-carbohydrate ratio (unfertilized plants). Unlike previous studies with mature wild grass species, we found that intact plants supported better performance than dried and ground plants, suggesting that cell wall removal may only improve performance for tougher or more carbohydrate-rich plants. These results add to the growing body of evidence suggesting that several migratory herbivorous species perform better on plants with a lower protein:carbohydrate ratio.

## 1 Introduction

Herbivores are often predicted to be limited by protein because plants typically have low nitrogen and, by extension, protein content relative to animals (1–4). However, herbivores with high energetic demands, such as migrating locusts, prefer and perform better when fed diets that are carbohydrate-biased (5–10). Most of these recent studies employed the Geometric Framework for Nutrition (GFN), which demonstrates consumers’ ability to balance dietary macronutrients like protein and carbohydrates. This regulation is key for maximizing performance (11). Thus, herbivores with high energetic demands are predicted to select plants with a low protein:carbohydrate ratio so they can meet their metabolic needs without overconsuming protein, as protein has been shown to have deleterious effects when consumed in high quantities (12–15). However, other factors such as mechanical and chemical defenses can influence host plant choice and herbivore performance. Here, we tested the interactive effects of host plant fertilization and wheat seedling state (intact or ground) on the preference and performance of the Australian plague locust, *Chortoicetes terminifera* (Walker) (Orthoptera: Acrididae), the most economically important and widespread locust species in Australia.

Locusts are grasshoppers that aggregate at high density and migrate long distances; they are also generalist herbivores (16). Long distance flights are fueled primarily by fat stores that are typically built *via* carbohydrate consumption (7, 17, 18). Indeed, locust outbreaks are often found in areas containing low protein, high carbohydrate plants, as has been shown in China (19), Australia (10, 20), and West Africa (8, 9, 21). When given the choice to balance dietary macronutrients with two complementary artificial diets using GFN methods, field populations of locusts select carbohydrate biased diets on which they have the highest growth and survival: *Oedaleus asiaticus* in China (5, 19), *Oedaleus senegalensis* in Senegal (8, 9), *Schistocerca cancellata* in Paraguay (7), and *Chortoicetes terminifera* in Australia (10, 22). Furthermore, *Oedaleus asiaticus* locusts fed their preferred protein:carbohydrate ratio fly for longer periods of time (5, 23). This pattern holds when eating plants. *S. cancellata* nymphs collected from marching bands preferred and gained more weight when fed plants with high carbohydrate contents (7). While *O. senegalensis* is found in environments where rapidly growing plants are often protein-rich, locusts are more numerous in fallow fields where soil fertility is lower (21) and plants contain more carbohydrates (9). This species preferred unfertilized over fertilized millet leaves, and had higher survival and laid heavier eggs when kept in field cages over unfertilized vs. fertilized millet (8). Collectively, these studies indicate that balancing protein and carbohydrate, and especially ensuring adequate carbohydrates, is an important factor influencing host plant choice for locusts. However, limited studies have investigated the interactive effects of plant nutrients and their mechanical properties on herbivory and on herbivores themselves.

In addition to raw nutrients, plant physical attributes, cell walls in particular, may restrict nutrient access and limit performance of herbivores (24, 25, 2004; 26). The plant cell wall is an extracellular matrix made of two main layers, the middle lamella and the primary cell wall, that encapsulate much of the plant cell’s nutritional content (27). The primary cell wall is made of cellulose which is undigestible, except by specialized consumers such as ruminants (28). Plant cell walls can hinder nutrient assimilation for herbivorous insects. For example, the Australian plague locust can only assimilate 40% of plant carbohydrate content when the cell wall is present compared to 90% when this barrier is mechanically overcome by grinding the plants (25). Surprisingly, insects were able to assimilate 80% of plant protein content when cell walls were intact (29). This suggests that carbohydrates are less accessible in some plant species than protein, although the mechanisms behind this are poorly understood. These studies were conducted using non-agricultural (undomesticated) grasses common to Australia and it is unknown if this pattern of inaccessibility holds for domesticated grass varieties that may be less defended mechanically and typically more protein-rich (25) or across agricultural regimes that may affect nutrient availability, such as soil amendments.

During outbreak years, the Australian plague locust, *C. terminifera*, invades rangeland and agricultural fields (30, 31), and wheat is grown on 42% of the 19.7 million ha of crop-growing land vulnerable to these outbreaks (32). We tested how locusts responded to wheat that was either fresh or ground and with different levels of nitrogen fertilizer. We predicted that fertilization would decrease, and grinding would increase, preference and performance by mechanically breaking down the cell wall and making carbohydrates more accessible. Understanding how both plant nutrient content and accessibility affect locust choice and performance when eating key crops is strategic for improving management programs for this serious pest.

## 2 Methods

### 2.1 Plant treatment and nutrient analysis

#### 2.1.1 Wheat treatments

We purchased seeds of red hard winter wheat (*Triticum aestivum L.*) from Sustainable Seed Company (South Salt Lake, Utah) and stored them in a freezer at −20°C until the beginning of the experiment. We chose this variety because of its hardiness and popular use as both a crop and as a dietary staple for lab-reared locust colonies. Wheat was grown hydroponically in a greenhouse at temperatures ranging from 20–22° C from November to January (light cycle 10.5 light hr:13.5 dark hr).

Seeds were first soaked for 18–24 hours in a cool dark area to initiate germination. We then placed 700–730 seeds in perforated containers (food-safe plastic, 16 × 13 × 4 cm) and covered them for two days. Once germinated, we placed those perforated containers in flood trays (Active Aqua AALR24B Low Rise Black Flood Table, ABS plastic, 121 × 61 × 13 cm). Every eight hours, each tray was flooded for 15 minutes. Three days after being placed into the flood trays, using the same watering regimen, the wheat for the fertilized treatment received 4.792 g.L^−1^ of urea (Greenway Biotech Inc. 46-0-0), an optimal amount for field-crop wheat (33). We dissolved the granulated urea in water and added to the sump of each hydroponic system. This fertilization period lasted three days to support optimal nitrogen uptake (34, 35). The control treatment received water for three weeks. For both control and fertilized treatments, we replaced the water used to flood the wheat every 4–5 days.

Because wheat and other cereal grains are most vulnerable to locust damage at the seedling stage, we used three-week old seedlings. Once plants had reached the desired age of 21 days old, we set half the wheat aside for the live-grass experiment, and cut the remaining half of both treatments down the base and dried the leaves at 60°C for 48 hours. Afterwards, we ground the dried wheat to particles of< 10 *μ* diameter (following 25) using a Retsch MM 400 ball mill at 30 Hz for 30 s. Ground leaves were then frozen at −20°C in airtight containers until use.

#### 2.1.2 Protein and carbohydrate analyses

Plant protein content was determined with a Bradford assay and the non‐structural carbohydrate content using the phenol‐sulphuric acid method on the dried and ground plant material (e.g., 22, 36).

### 2.2 Australian plague locust and experimental design

#### 2.2.1 Locusts

Our *C. terminifera* lab colony is hosted at Arizona State University (Arizona, USA) and was established in 2015 from a colony originating from The University of Sydney (New South Wales, Australia). The Australian lab colony was started with wild locusts collected in 2005 and 2006 from Eastern and Western Australia (37) and has since been supplemented with field locusts in 2017. The ASU colony is reared on a 14 h light:10 h dark cycle with RH = 20–50%, and a 30 ± 2 °C (light) 25 ± 2 (dark) °C daily temperature cycle. Locust colonies are fed non-fertilized hydroponically grown wheat seedlings, supplemented with wheat bran (Tempe Feed) treated with tri-sulfa (Sigma Aldrich) for colony health.

#### 2.2.2 Fresh wheat: choice experiment

**Figure 1:**
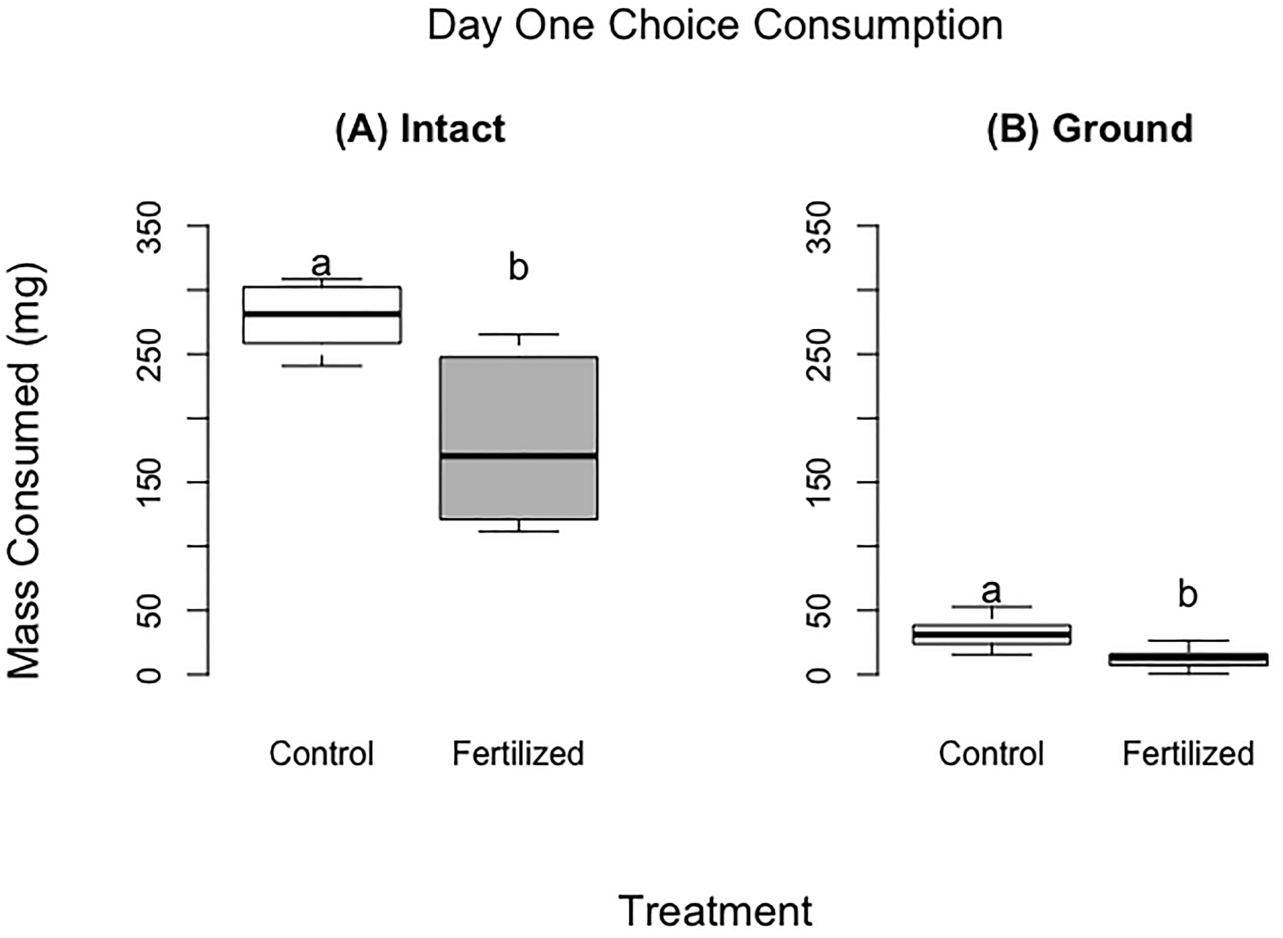
Food consumed for the choice experiments after 24 hours for the fresh wheat experiment (panel **A**) and for the ground wheat (panel **B**). There were 60 locusts per treatment for panel **(A)** and 13 locusts per treatment for panel **(B)** Different letters indicate significant differences of p<0.05 between groups. Boxplots show medians and interquartile ranges.

All experiments started when locusts molted into their last nymphal instar (5th instar) at which point they were weighed, sexed, and placed in an experimental enclosure. In total we used 12 wire mesh cages (45 cm long × 45 cm wide × 45 cm tall), each containing ten individuals (five males and five females). We did this to more accurately measure the amount of consumption as the individual consumption measurements would be more prone to inaccuracy due to small differences in amount consumed. The food source needed to be cut into “patties” containing wheat sprouts still connected to bare roots to remain turgid throughout the duration. We fed each cage of locusts two clipped and pre-weighed wheat patties presented in 8 × 6 cm food containers (**SI**, **Figure 1**). One food container was filled with nitrogen-fertilized wheat and the other with unfertilized (control) wheat. After 24 hours, locusts were removed and weighed. The remaining wheat was dried for 48 hours and weighed to measure consumption. This experiment used a group because there was more than one food source and there would have been major logistic issues with to keeping track of individual consumption on multiple foods. We estimated dry consumption using a regression equation linking the mass of fresh wheat to the mass of dried wheat. For this, we recorded the mass of 15 patties of control wheat and 15 patties of fertilized wheat, dried them for 48 hours at 60° C, and recorded their dried mass. The regression equation is presented in the Supporting Information (**SI**, **Table 1**).

**Table 1:** Results from a MANOVA comparing the protein and carbohydrate contents (%) between fertilized and control (unfertilized) wheat plants.

*Variable*	*Source*	*df*	*F-ratio*	*p-value*
*Carbohydrate & Protein content (%)*	Treatment	1	72.16	<0.0001*
*Carbohydrate content (%)*	Treatment	1	109.32	<0.0001*
*Protein content (%)*	Treatment	1	0.02	0.89
*Total Macronutrient (%)*	Treatment	1	109.32	<0.0001*

Results from ANOVAs comparing carbohydrate content (%), protein content (%), and total macronutrient content (%) between fertilized and control (unfertilized) wheat plants.

#### 2.2.3 Fresh wheat: no-choice experiment

For the no-choice experiment, we used ten cages per treatment (fertilized and control). Each cage contained six individuals (three females and three males) that were individually marked on the pronotum with Sharpie brand (Atlanta, Georgia) paint markers. We used six individuals per cage for this experiment because that number allowed us to better measure fresh mass consumed of a single plant choice. We replaced the wheat patty every day until the locusts molted or died. We recorded locust mass and frass production (mg) every three days, as well as development time. We recorded consumption for days 0–3, days 0–6, and total consumption; locust body mass change for days 0–3, days 0–6, and total locust body mass change; development time (the duration of the locusts’ fifth stadium); survival; total frass production; and assimilation. Assimilation was calculated using the following formula:


$$Assimilation\;=\frac{\;Mass\;of\;wheat\;consumed\;by\;cage\;-\;Mass\;of\;frass\;produced\;by\;cage\;}{Mass\;of\;wheat\;consumed\;by\;cage}$$

#### 2.2.4 Ground wheat: choice experiment

We placed 26 freshly molted 5^th^ instar locusts (half males and half females) into individual 17.5 × 11.5 × 4.5 cm perforated polystyrene cages with a perch for roosting and a water tube. Each cage contained two pre‐weighed dishes: one filled with fertilized and the other control (unfertilized) ground wheat. After three days, we removed any frass present in the dish and dried the diets for 24–36 hours at 60° C and then weighed the diets to measure the amount of ground wheat consumed.

#### 2.2.5 Ground wheat: no-choice experiment

The no-choice setup was identical to the choice experiment, except that a locust received only one food dish per cage (fertilized ground wheat or control ground wheat). We used 26 individually housed locusts per treatment. We then removed the diet dishes after three days, and dried, weighed and replaced the dishes with new pre-weighed dishes. The no-choice experiment ended when the locusts molted or died. We recorded consumption for day 0–3, day 0–6, total consumption, locust mass change for day 0–3, day 0–6, day 0–9, total locust mass change, development time and survival, frass production, and food assimilation.

#### 2.2.6 Artificial diet vs ground wheat: no-choice experiment

This experiment was run to test if dry foods, those with a complete lack of edible water, was to blame for high mortality and poor molting success found in other experiments. To compare the effects of dried, ground wheat to a dried and powdered artificial diet containing all nutrients needed for locust growth and development, we ran a final experiment. An experiment similar to those with ground no-choice tests but instead of comparing the performance of wheat treatments, we compared the performance of control wheat to an artificial diet using ratios selected by field populations of *C. terminifera* (p14:c28) (10). We used 16 locusts (8 for each treatment; 1:1 sex ratio) and followed the same protocol as described in previous sections.

### 2.3 Statistical analyses

Prior to any statistical analysis, we assessed all data collected for normality and homoskedasticity, which we found to be true. To compare protein and carbohydrate contents between fertilized and control wheat, we performed a MANOVA. For all experiments, we analyzed consumption and locust mass change using ANCOVAs with locust initial mass as a covariate to account for size differences and sex as a cofactor. For development time and survival, Kaplan-Meier survival analyses were used. For both the ground and the fresh grass experiments, we calculated frass and consumption rates (e.g., consumption/days in experiment) and analyzed both using ANCOVA’s and locust initial mass as a covariate. For all analyses besides the survival analysis, locusts that were not alive for the duration of the interval recorded (e.g. day 0–3 or day 0–6) were removed from the analysis. We presented the cumulative results for standardized time periods (days 0–3 and days 0–6) as well as for the whole experiment (day 0 to time of molt). All statistical analyses were conducted using R studio version 1.3.1073. as well as JMP Pro 15.2.0

## 3 Results

### 3.1 Protein and carbohydrate content of wheat plants

The fertilization treatment significantly increased the protein:carbohydrate ratio of wheat plants expressed in %p: %c of dry mass, from p28:c14 (control) to p29:c8 (fertilized) (**Table 1**) (F=72.16, P<0.001). This pattern was driven by a decrease in carbohydrate content from 14.24% ± 1.78 SE in control plants to 7.52% ± 0.52 SE in fertilized plants (F=109.32, P<0.001). There was no significant effect of fertilizer on protein content; the average for across both treatments were 28.13% ± 3.89 SE (F=0.02, P = 0.89). Fertilization decreased total macronutrient content from 41.86% ± 8.25 SE to 36.15% ± 1.84 SE (F =109.32, P<0.001).

### 3.2 Choice experiments: insights into preference

**Figure 2:**
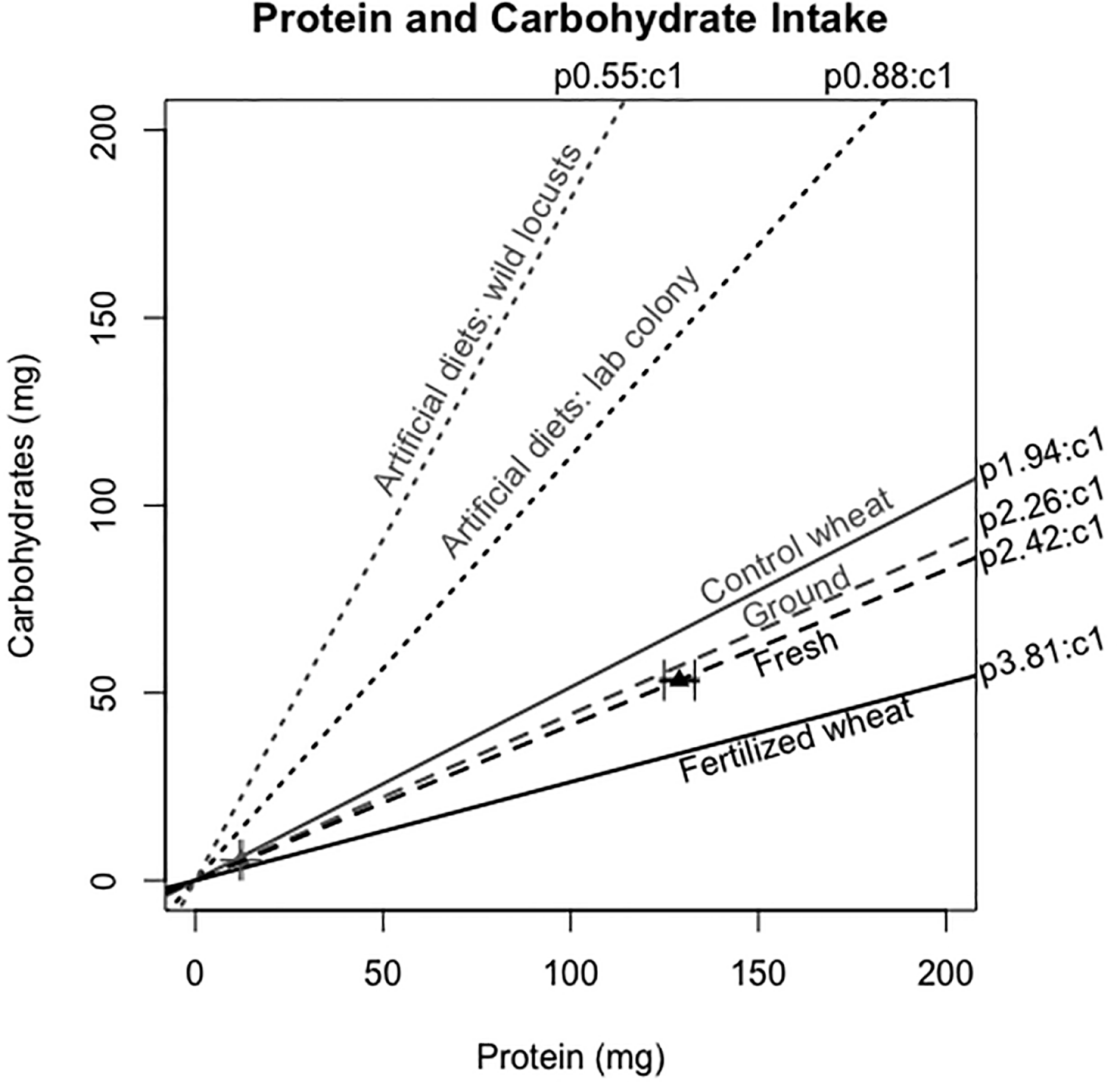
Protein:carbohydrate intake (p:c intake) of locusts when presented with a choice between fertilized and control wheat in fresh (gray dashed line) and ground (black dashed line). The dotted lines represent their p:c intakes and the triangles indicate the raw means and their standard errors of the mean (SEM's) of the amount consumed after 24 h. The solid lines represent the p:c ratio of the fertilized and control wheat plants. There were 60 locusts per treatment for the fresh wheat experiment and 13 locusts per treatment for the ground wheat experiment. For comparison, we have added results from separate studies showing the preferred p:c ratio from a field population (10) and a lab colony (37) measured using artificial diets with a broad range of accessible p:c spanning 7p:35c to 35p:7c.

Locusts that were provided with fresh wheat consumed 1.75 times the control wheat compared to fertilized wheat, by dry mass (**Table 2**, **Figure 1**) (F =30.45, P<0.001). Similarly, locusts placed on the ground wheat treatments ate 2.9 times more of the control wheat than the fertilized wheat. (**Table 2**, **Figure 1**) (F =60.08, P<0.001). For both ground and fresh plant experiments, the locusts selected very similar ratios of protein to carbohydrates (p28:c11 fresh vs p28p:c12 ground) (**Figure 2**).

**Table 2:** ANCOVA results comparing the consumption (mg) between fertilized and control (unfertilized) wheat for the two choice experiments.

*Variable*	*Source*	*df*	*F-ratio*	*p-value*
*Fresh Consumption (mg)*	Treatment	1	30.45	<0.0001*
	Start Mass (mg)	1	7.01	0.02*
*Ground Consumption (mg)*	Treatment	1	60.08	<0.0001*
	Start Mass (mg)	1	2.00	0.16

Treatment refers to the wheat (fertilized or control). Locust wet start mass was used as a covariate to adjust for size differences among insects. For the fresh wheat experiment we used 12 replicates and 10 grasshoppers per replicate. For the ground treatment we used 26 replicates and 1 grasshopper per replicate.

### 3.3 No choice experiments: insights into performance

#### 3.3.1 Consumption, locust mass change, frass production, and assimilation on fresh wheat

**Figure 3:**
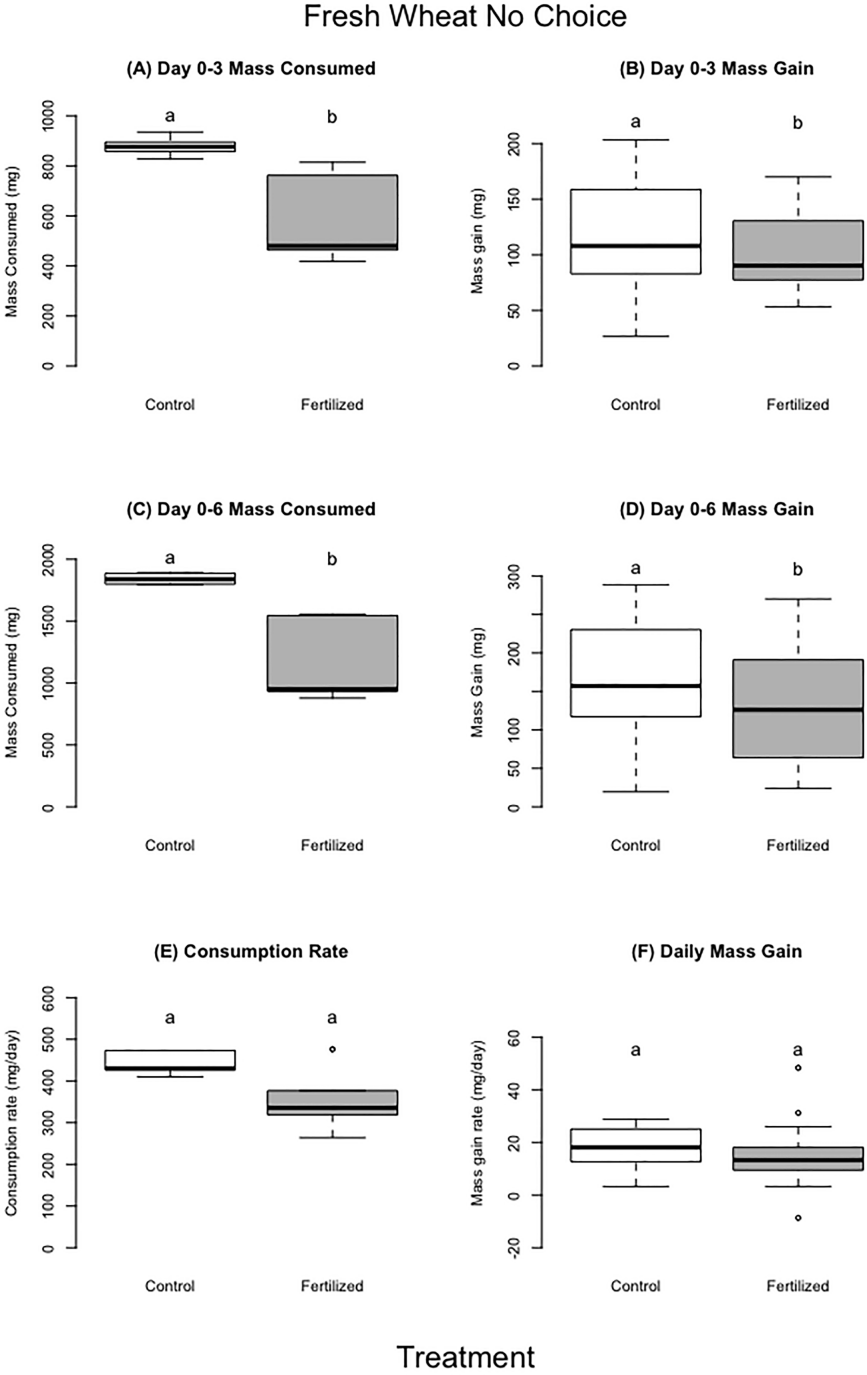
Fresh wheat no choice experiment. Panel **A, C** represent average amount of food consumed by locusts for each treatment for days 0–3 (panel **A**) and days 0–6 (panel **C**). Panel **(B, D)** represent the mass gain by locusts for each treatment on day 0–3 (panel **B**), days 0–6 (panel **D**). Panels **(E, F)** show the consumption (panel **E**) or mass gain (panel **F**) daily rate of consumption corrected for individual’s time in experiment. For each treatment we used 10 replicates with 6 grasshopper per replicate. There were 60 locusts in experiment for day 0–3, and 30 locusts in the experiment for day 0–6 (individuals not included either molted or died). Different letters indicate significant differences of p<0.05 between groups. Boxplots show medians and interquartile ranges, with any outliers represented as open circles.

**Figure 4:**
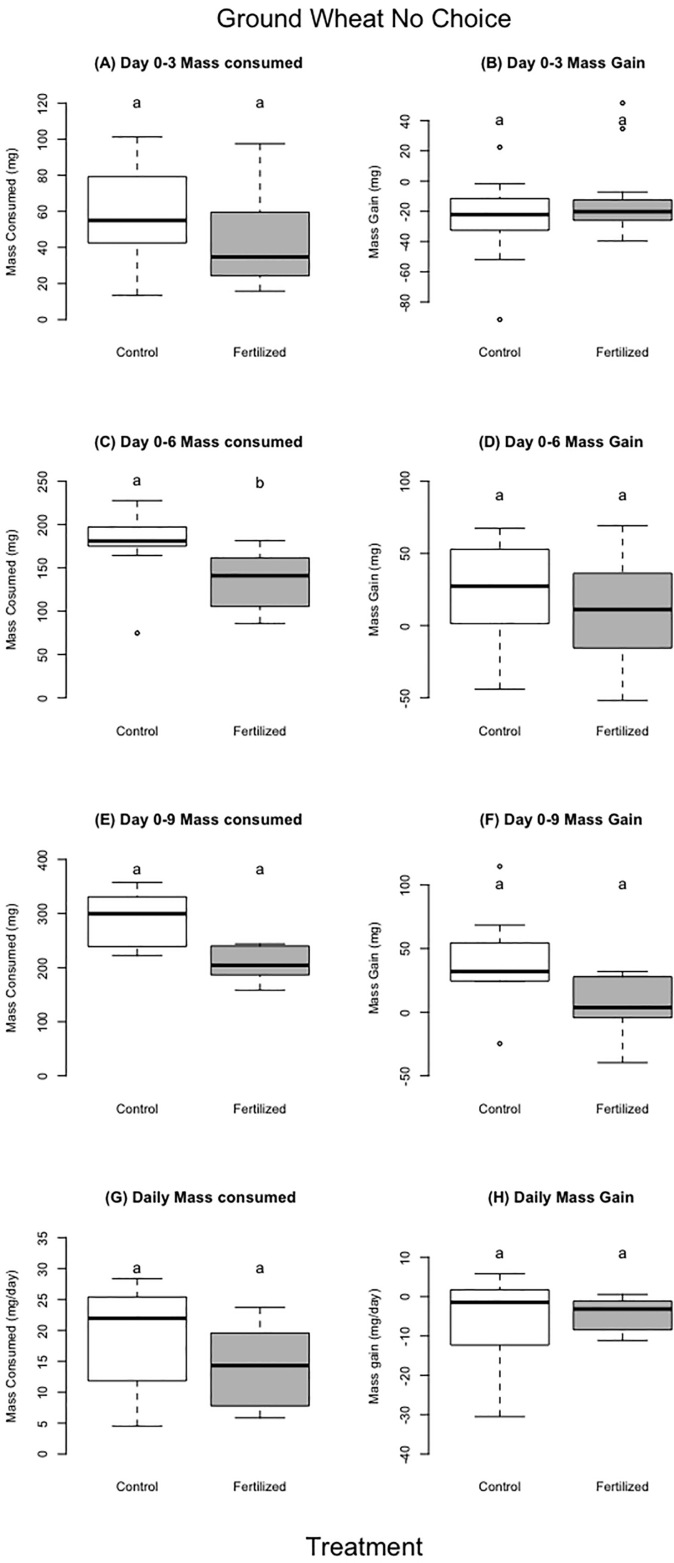
Dried ground wheat no choice experiment. The left column (**A**, **C**, **E**, and **G**) shows food consumed and the right column (**B**, **D**, **F**, **H**) shows mass gain over different time periods in the experiment. For each treatment we used 13 individual locusts per replicate. There were 26 locusts in the experiment for day 0–3, and 18 locusts in experiment for day 0–6 (individuals that were removed either molted or died) and 11 for days 0–9. Different letters indicate significant differences of p<0.05 between groups. Boxplots show medians and interquartile ranges, with any outliers represented as open circles.

The locusts in the control treatment consumed more wheat than those fed fertilized wheat during the first three days and up to six days (~400 mg more, 27% more by dry mass) (F=8.93, p= 0.02;F=7.78, p=0.03 respectively), but there were no significant differences for the entirety of the experiment (**Table 3**; **Figure 3**) (F=2.52, P=0.16). Locusts from the control treatments gained more weight in the first three days and up to six days compared to locusts fed the on fertilized wheat (**Table 3**, **Figure 3**) (F=4.29, P= 0.04;F=4.13, P=0.05 respectively), but there was no difference in mass gain for the duration of the whole experiment (F=2.26, P=0.14). Locusts produced more frass when fed wheat from the control treatment than nitrogen-amended (fertilized) wheat (**Table 3**; **Figure 4**) (F=31.88, P= 0.001), however assimilation was not significant (**Table 3**; **Figure 4**) (F=5.50, P=0.06).

**Table 3:** Results from no-choice fresh plant experiments for consumption (mg), mass gain, frass production (mg), and assimilation from ANCOVAs with initial body mass as a covariate and sex as a cofactor.

*Variable*	*Source*	*df*	*F-ratio/ChiSq*	*p-value*
*Consumption Day 0–3 (mg)*	Treatment	1	8.93	0.02*
	Start Mass (mg)	1	0.003	0.96
	Sex	1	13.24	0.01*
*Consumption Day 0–6 (mg)*	Treatment	1	7.78	0.03*
	Start Mass (mg)	1	0.003	0.96
	Sex	1	19.00	0.005*
*Consumption Rate (mg/day)*	Treatment	1	2.52	0.16
	Start Mass (mg)	1	5.52	0.06
	Sex	1	2.49	0.17
*Mass Gain Day 0–3 (mg)*	Treatment	1	4.29	0.04*
	Start Mass (mg)	1	3.59	0.06
	Sex	1	68.73	<0.0001*
*Mass Gain Day 0–6 (mg)*	Treatment	1	4.13	0.05*
	Start Mass (mg)	1	19.21	<0.0001*
	Sex	1	57.19	<0.0001*
*Mass Gain Day 0–End (mg/day)*	Treatment	1	2.26	0.14
	Start Mass (mg)	1	45.63	<0.0001*
	Sex	1	129.89	<0.0001*
*Frass Production (mg/day)*	Treatment	1	31.88	0.001*
	Start Mass (mg)	1	1.06	0.34
	Sex	1	24.36	0.003*
*Assimilation (%)*	Treatment	1	5.50	0.06
	Start Mass (mg)	1	2.37	0.17
	Sex	1	0.29	0.61
*Molting Rate*	Treatment	1	0.44	0.51
*Death Rate*	Treatment	1	0.17	0.68

Molting and survival rates were compared using Kaplan Meier survival analyses. Treatment refers to the wheat (fertilized or control). For each treatment we used 25 replicates with one grasshopper per replicate. There were 59 locusts alive per treatment for day 0–3, and 37 locusts alive per treatment for day 0–6.

#### 3.3.2 Consumption, locust mass change, frass production, and assimilation on ground wheat

**Figure 5:**
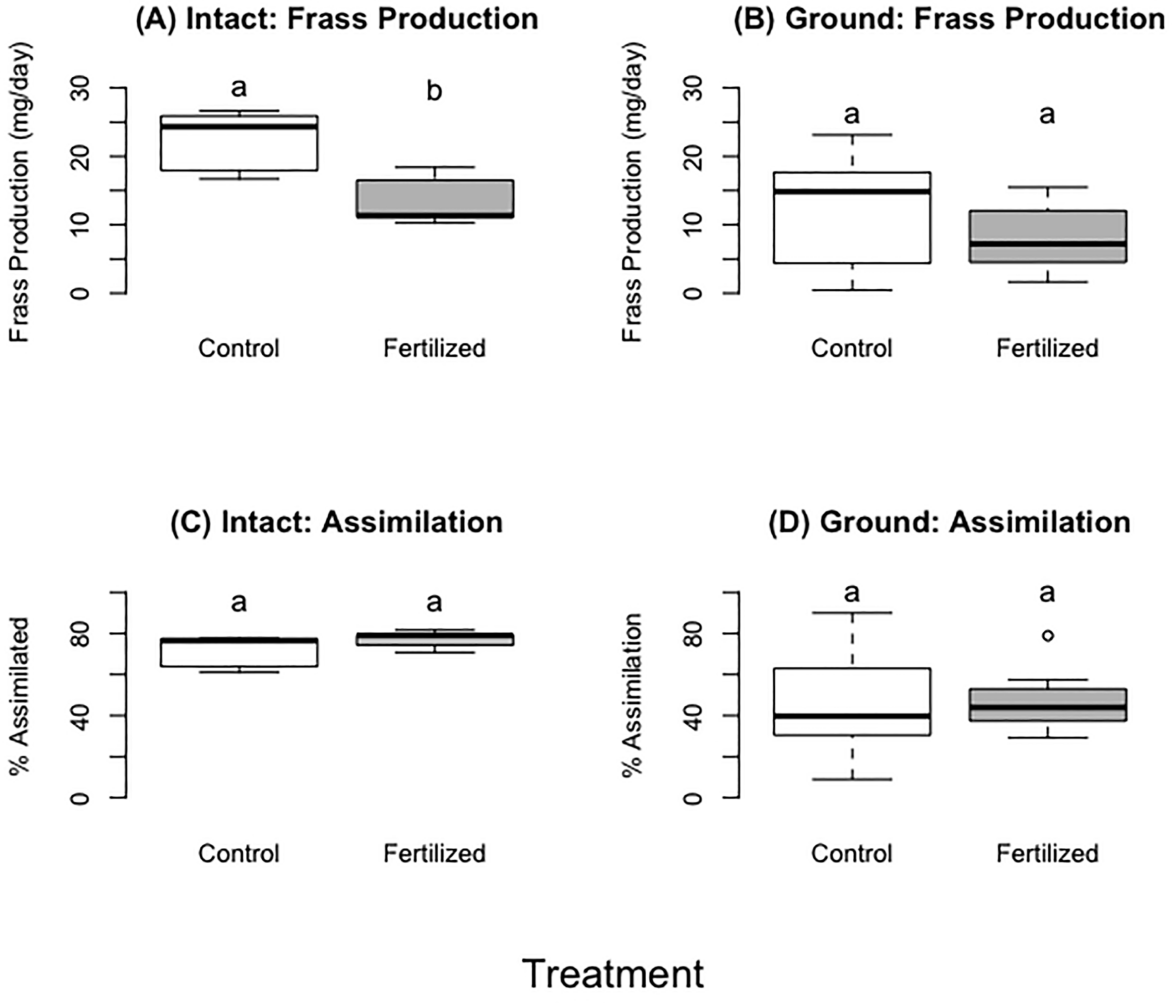
**(A, B)** showing frass production of fresh plants **(A)** or dried ground plants **(B)**. **(C, D)** showing food assimilation when locusts consumed fresh **(C)** or dried ground plants **(D)**. Different letters indicate significant differences of p<0.05 between groups. Boxplots show medians and interquartile ranges, with any outliers represented as open circles.

The locusts in the control treatment, feeding on ground unfertilized wheat, consumed more food than locusts fed the ground fertilized wheat for the first six days; there were no differences for days 0–3, days 0–9, or for the entire experiment (**Table 4**, **Figure 5**). There was no difference in locust mass change for any of the aforementioned time periods (**Table 4**, **Figure 5**). There was also no difference between control and fertilized treatments for frass production (**Table 4**), or assimilation (**Table 4**).

**Table 4:** Results from the no-choice ground plant experiments for consumption (mg), mass variation (mg), frass production (mg), and assimilation from using ANCOVAs with start mass as a covariate and sex as a cofactor.

*Variable*	*Source*	*df*	*F-ratio/ChiSq*	*p-value*
*Consumption Day 0–3 (mg)*	Treatment	1	1.40	0.25
	Start Mass (mg)	1	2.17	0.15
*Consumption Day 0–6 (mg)*	Treatment	1	4.97	0.04*
	Start Mass (mg)	1	0.48	0.50
*Consumption Day 0–9 (mg)*	Treatment	1	12.2	0.004*
	Start Mass (mg)	1	0.18	0.67
*Consumption Day 0–End (mg/day)*	Treatment	1	0.18	0.67
	Start Mass (mg)	1	0.13	0.72
*Mass Gain Day 0–3 (mg)*	Treatment	1	1.76	0.20
	Start Mass (mg)	1	1.72	0.20
*Mass Gain Day 0–6 (mg)*	Treatment	1	0.59	0.46
	Start Mass (mg)	1	0.94	0.34
*Mass Gain Day 0–9 (mg)*	Treatment	1	3.49	0.09
	Start Mass (mg)	1	1.29	0.27
*Daily Mass Gain (mg/day)*	Treatment	1	1.85	0.19
	Start Mass (mg)	1	3.30	0.08
*Frass Production (mg/day)*	Treatment	1	1.71	0.22
	Start Mass (mg)	1	0.18	0.67
*Assimilation (%)*	Treatment	1	0.01	0.94
	Start Mass (mg)	1	1.36	0.26
*Molting Rate*	Treatment	1	0.16	0.69
*Death Rate*	Treatment	1	1.35	0.25

Molting and survival rates were compared using Kaplan Meier survival analyses. Treatment refers to the wheat (fertilized or control). For each treatment we used 25 replicates with one grasshopper per replicate. There were 22 locusts alive per treatment for day 0–3, 16 locusts alive for day 0–6, and 15 locusts alive for day 0–9.

### 3.4 Survival and molting success of locusts on fresh and ground wheat

**Figure 6:**
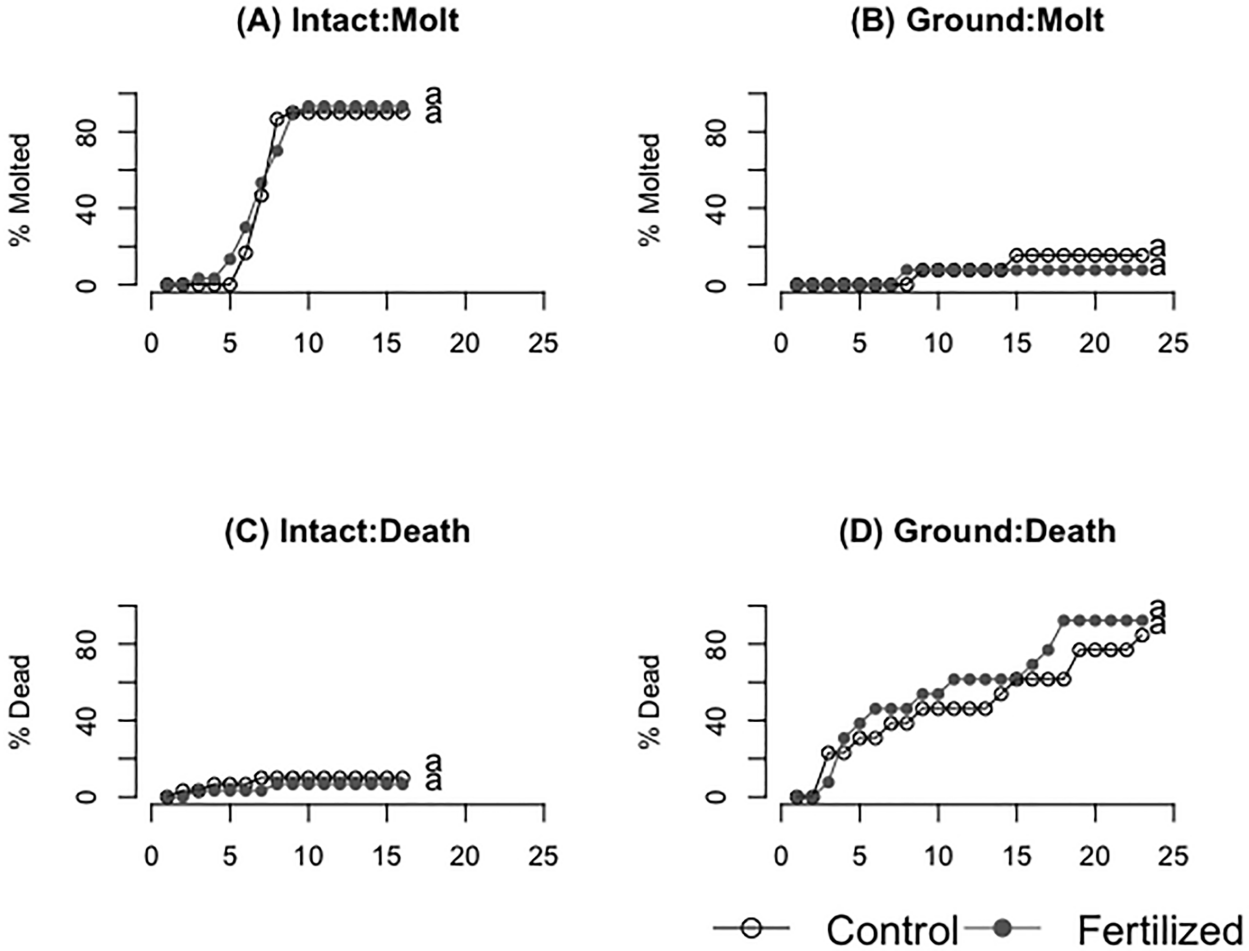
**(A, B)** show molting success of fresh **(A)** and ground **(B)** wheat treatments respectively. **(C, D)** show the mortality of locusts consuming fresh **(C)** and ground **(D)** wheat treatments respectively. Different letters indicate significant differences of p<0.05 between groups.

There was high molting success for the locusts eating fresh plants and meager molting success for locusts eating dried ground plants (**Figure 6A, B**, **Table 3**, **4**) but there was no significant difference between the control and fertilized groups within each experiment (fresh: F=0.44 P=0.51 dried ground: F= 0.16, P=0.69). There were similarly no statistical differences in death rate for the two experiments between the control and fertilized groups within each experiment, but there was a higher death rate in those eating dried ground grass (**Figure 6C, D**, **Table 3**, **4**) (fresh: F=0.17 P=0.68 dried ground: F= 1.35, P=0.25).

### 3.5 Artificial vs control ground wheat: consumption, locust mass change, and survival

**Figure 7:**
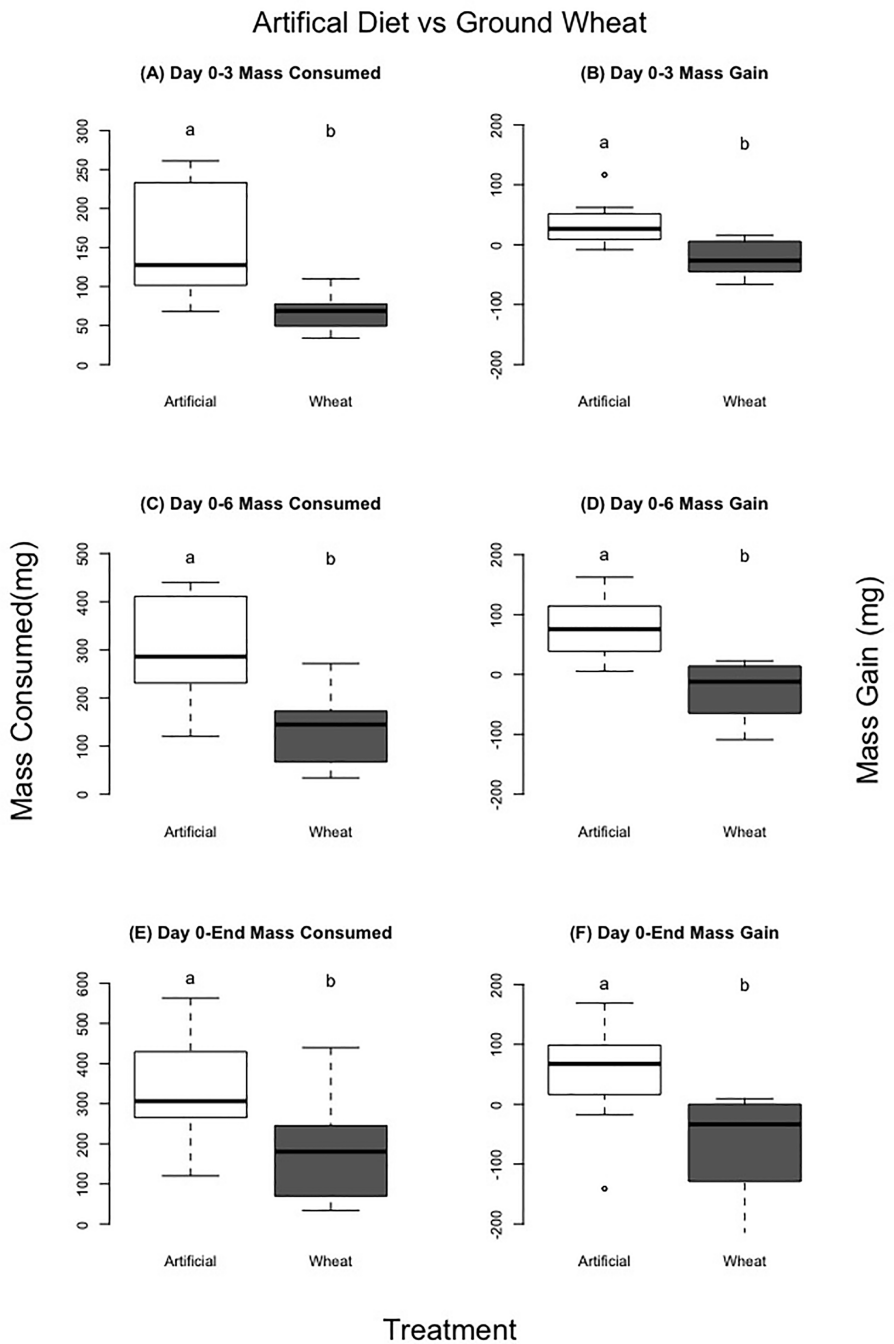
Comparison of no choice experiments for locusts eating artificial diets vs control dried ground wheat grass. Left column **(A, C, E)** compares mass gained, right column **(B, D, F)** mass consumed over different time periods in the experiment. Different letters indicate significant differences of p<0.05 between groups. Boxplots show medians and interquartile ranges, with any outliers represented as open circles.

**Figure 8:**
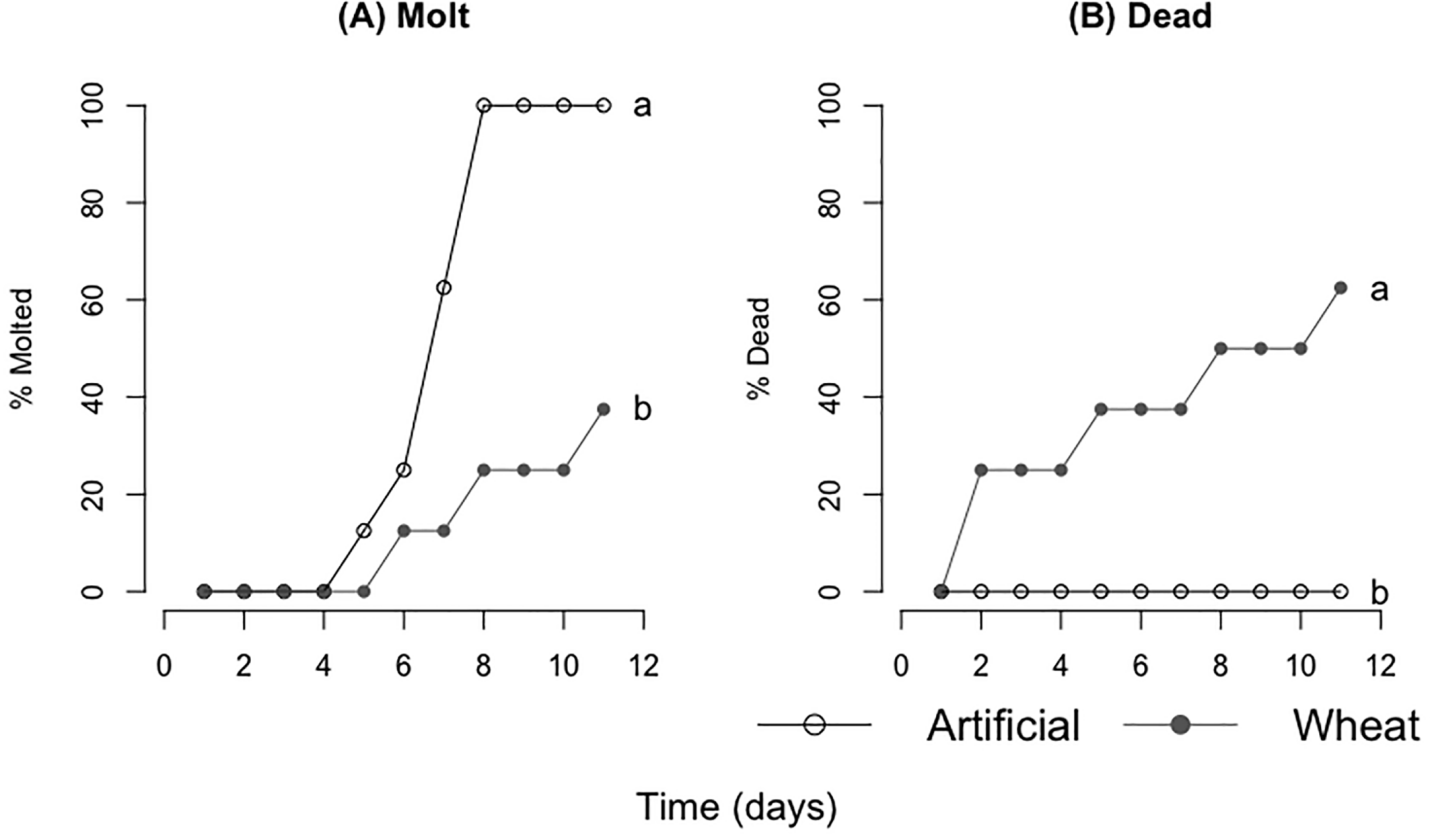
Survival analyses comparing **(A)** cumulative molts and **(B)** cumulative deaths. Ground artificial diet is depicted as the open circles with the accompanying line and control ground wheat is depicted as the filled gray circles with the accompanying line (8 locusts per treatment). Different letters indicate significant differences of p<0.05 between groups.

There was a significant difference in both mass consumed and locust mass change between the two groups; the artificial diet group consumed and acquired more mass for each time interval. For days 0–3, 0–6, and from day 0–End (**Table 5**, **Figure 7**) (mass consumed: F=10.52, P=0.01; F=9.74, P=0.01;F=2.47, P=0.03) (mass gained: F=15.86, P=0.01; F=14.00, P=0.01; F=2.45, P=0.03). Locust mortality was significantly different between treatments (molt rate:F=4.35, P=0.4; Death rate: F=11.13, P=0.008). Locusts fed the artificial diet molted in about 7 days and had no mortality, while those fed the control wheat molted after about 9 days and had 60% mortality (**Table 5**, **Figure 8**).

**Table 5:** Results from the no-choice ground vs artificial experiments for consumption (mg), mass variation (mg), frass production (mg), and assimilation from using ANCOVAs with start mass as a covariate.

Variable	Source	df	F-ratio/ChiSq	p-value
*Mass Gain Day 0–3 (mg)*	***Treatment***	***1***	***10.52***	0.01*
	***Start Mass (mg)***	***1***	***0.80***	***0.39***
*Mass Gain Day 0–6 (mg)*	***Treatment***	***1***	***9.84***	0.01*
	***Start Mass (mg)***	***1***	***0.39***	***.55***
*Daily Mass Gain (mg/day)*	***Treatment***	***1***	***6.12***	0.03*
	***Start Mass (mg)***	***1***	***.05***	***.8243***
*Consumption Day 0–3 (mg)*	***Treatment***	***1***	***15.86***	<0.01*
	***Start Mass (mg)***	***1***	***6.96***	.02*
*Consumption Day 0–6 (mg)*	***Treatment***	***1***	***14.00***	<0.01*
	***Start Mass (mg)***	***1***	***2.68***	***0.13***
*Consumption Rate (mg/day)*	***Treatment***	***1***	***6.02***	0.03*
	***Start Mass (mg)***	***1***	***2.42***	***0.14***
*Molting Rate*	***Treatment***	***1***	***4.35***	0.04*
*Death Rate*	***Treatment***	***1***	***11.13***	0.0008*

Molting and survival rates were compared using Kaplan Meier survival analyses. Treatment refers to the food (artificial or control wheat). For each treatment we used 16 replicates with one grasshopper per replicate.

## 4 Discussion

Growing evidence indicates that final instar locusts prefer and perform best on diets with a lower protein to carbohydrate ratio, whether feeding on artificial diets or plants (reviewed in 6), and this study provides some support for that pattern. Locusts increased consumption on control wheat treatment, which had both a higher caloric and carbohydrate density. This increase in consumption corresponded with an increase in mass, likely as lipid (7). However, besides growth, this increase in food consumption did not improve nutrient assimilation, molting success, or survival. In contrast to previous studies (25, 29), we did not find that breaking down plant cell walls increased locust performance. In fact, locusts performed better on intact wheat than they did on dried ground wheat, which may have been due to mechanical or nutrient differences (38, 39) in the grass species used and/or how plants were dried. This research question could benefit from further investigation.

When locusts are given the choice between foods differing in macronutrient content, they will self-regulate by compensatory feeding of each plant to strategically meet their ideal p:c ratio (6). Locusts and swarming grasshoppers tend to select a carbohydrate-biased p:c intake target (6). For example, final (5^th^) instar *C. terminifera* selected 1p:1.13c in a lab population (37) and 1p:1.8c to 1p:1.37c in field populations (10). However, protein-biased nutritional landscapes are common in agricultural settings and for young rapidly growing plant tissues (8, 40). Indeed, both control and fertilized wheat in our study contained more protein than carbohydrates: control was p1.94:c1 and fertilized was p3.81:c1 (**Figure 2**). Thus, locusts in our study were constrained to a protein-biased diet for both plant choice and plant no-choice experiments. Accordingly, the self-selected p:c ratios in the choice experiment were both protein-biased, albeit slightly less protein-biased for the dried ground wheat (2.42p:1c vs. p2.26:c1).

Results from all experiments indicated that locusts preferred the control wheat over the fertilized wheat. In choice experiments, locusts ate about 2–3 times the amount of control relative to fertilized wheat; in no choice experiments, locusts ate about 30–70% more over 6 days when confined to control wheat relative to fertilized. Locusts conferred some benefits in eating the lower p:c wheat. In fresh wheat experiments, locusts eating control wheat had a faster weight gain in the first 6 days, though there was not a significant effect of fertilization on food assimilated or on molting or death rate. Our study was conducted on a single nymphal stadium and it is likely that longer-term experiments would show stronger effects. For example, a long term study on caterpillars (*Heliothis virescens*) showed that there is only a narrow range of p:c that maximizes performance over the course of their lifespans (41). Because balancing p:c intake is a primary driver of foraging behavior and growth for insect herbivores (42), it is likely the lower p:c ratio of control plants increased preference and growth rate. Given that protein amounts were similar in control and fertilized wheat plants (**Table 1**), our results suggest that carbohydrates may be a key factor in this choice. Locusts may have chosen plants based on total energy content as unfertilized plants were more macronutrient dense (36.15% ± 1.84 SE and 41.86% ± 8.25 SE, respectively). It is also possible that using a simple N fertilizer like urea decreased micronutrients; other studies have shown (43) usage of urea can cause a negative effect that producers may avoid by using fertilizers with more micronutrients like aminochelates. Nevertheless, our results corroborate other regional studies (China (5, 2012), Australia (10, 2020), and West Africa (8, 9, 21, 44)) indicating that late-instar locust and swarming grasshopper species prefer and perform best on low p:c diets.

Our expectation was that grinding wheat to particles smaller than 10*μ*m would increase nutrient accessibility, particularly soluble carbohydrates, and therefore increase performance. Another study on 5^th^ instar *C. terminifera* nymphs found that grinding freeze dried Mitchell grass (*Astrebla lappacea*) improves nutrient accessibility and assimilation (25). Furthermore, this same study (25) showed that locusts were able to extract 50% more carbohydrates when plant cell walls were removed by grinding than when they were consuming fresh plants (25). Therefore, we expected preference for unfertilized wheat (less protein-biased plant) to be more pronounced when using intact plants since carbohydrates should be harder to access. However, we found similar preference for unfertilized plants in both fresh and dried ground studies. Moreover, for locusts fed dried ground plants in our study, we recorded lower food consumption, less successful molts to adult, and lower survival.

We have identified potential factors that may explain the differing effects of dried ground plants on locusts between Clissold et al. (25) and our study. In the Clissold et al. (25) study, lyophilizing (freeze drying) the plants instead of desiccating them in a drying oven may have helped maintain nutrient and vitamin levels that may have been degraded through the drying process in our samples. Another potential explanation may lie in plant structure as leaf toughness can affect nutrient accessibility and plant choice (25, 29, 45). Here we used a seedling (3-4 weeks old) cereal crop, wheat, which is not as thick as the wild Mitchell grass (*A. lappacea*) used in the Australian study (12-14 weeks old). Thus, grinding the tough Mitchell grass may have released more nutrients than grinding soft wheat sprouts. Grasses (Poaceae) can contain high concentrations of silica (46) which can wear out herbivore mandibles and decrease consumption (47, 48). Silica has been shown to deter locusts which prefer to eat plants with lower silica concentration (49–52). It is possible that grinding the plants released silica structures inside the gut of the insects, causing internal damage (51, 53), though that would not explain higher performance in locusts eating ground grass in the Clissold et al. (25) study. A final explanation may be major differences in nutritional content between these two plant species. Adult Mitchell grass contains 9.7% protein and 24.1% carbohydrates (25) while our seedling unfertilized wheat plants contained 27.6% protein and 14.2% carbohydrates. Thus, grinding wheat seedlings may not have released the same amount of soluble carbohydrates. More studies using additional combinations of plant and grasshopper species are needed to disentangle the relative importance of these factors.

We contrasted experiments using control dried ground plants with dry powder artificial diets to ensure low consumption and growth rates were not due to diets being a dry powder. Locusts eating the artificial diet had substantially higher consumption and growth rates than locusts eating dried ground plants. This result may be partially due to the p:c ratio of the artificial diet (14p:28c) being a better match to the preferred p:c of *C. terminifera* populations (10, 37), which likely further supported improved growth. However, rates for the artificial diet overlapped with consumption and growth rates for locusts eating control fresh wheat, so there was likely a combination of nutritional and structural factors at play.

In conclusion, we showed that fertilizing young wheat makes it less preferred by Australian plague locusts than control wheat, and that locusts will decrease consumption if confined to fertilized wheat seedlings. It was important to test young plants because seedling stages are typically more vulnerable to locust attacks on foliage since older plants with more leaves can better tolerate herbivory (32, 54). While seedling wheat crops regularly sustain considerable damage from *C. terminifera* in Australia (32), we showed that their nutritional profile is far from the optimal p:c ratio for this locust species. Our results suggest that carbohydrate is potentially a limiting nutrient for *C. terminifera*, particularly in agricultural settings where nymphal bands attack seedling plants that may be richer in protein than what is usually assumed for grasses. This study represents an important step in bridging the gap between theoretical knowledge developed using artificial diets and practical advances that can form the basis of a nutritionally-based management program for herbivorous pests.

## Data availability statement

The data presented in the study are deposited in the Dryad repository, https://datadryad.org/stash/dataset/doi:10.5061/dryad.5hqbzkh9d. Further inquiries can be directed to the corresponding author.

## Author contributions

JB and MG contributed to the conceptualization and design of the study. JB and SM collected the data, and JB organized the database. JB and MG performed the statistical analysis. JB made figures and tables. JB and MG wrote the original manuscript. MG administrated the project and supervised it. AC and RO acquired funding and provided the resources necessary to complete the project. All authors contributed to the revisions of the manuscript. All authors read and approved the submitted version.
