# Supplementary material for: Nutrient supply and accessibility in plants: effect of protein and carbohydrates on Australian plague locust (Chortoicetes terminifera) preference and performance
Source: Front Insect Sci. 2023 Jul 13;3:1110518. doi: 10.3389/finsc.2023.1110518 (PMC10926423; doi:10.3389/finsc.2023.1110518)
Supplement: Supplementary file 1 [file DataSheet_1.docx]

*Supplementary Information*


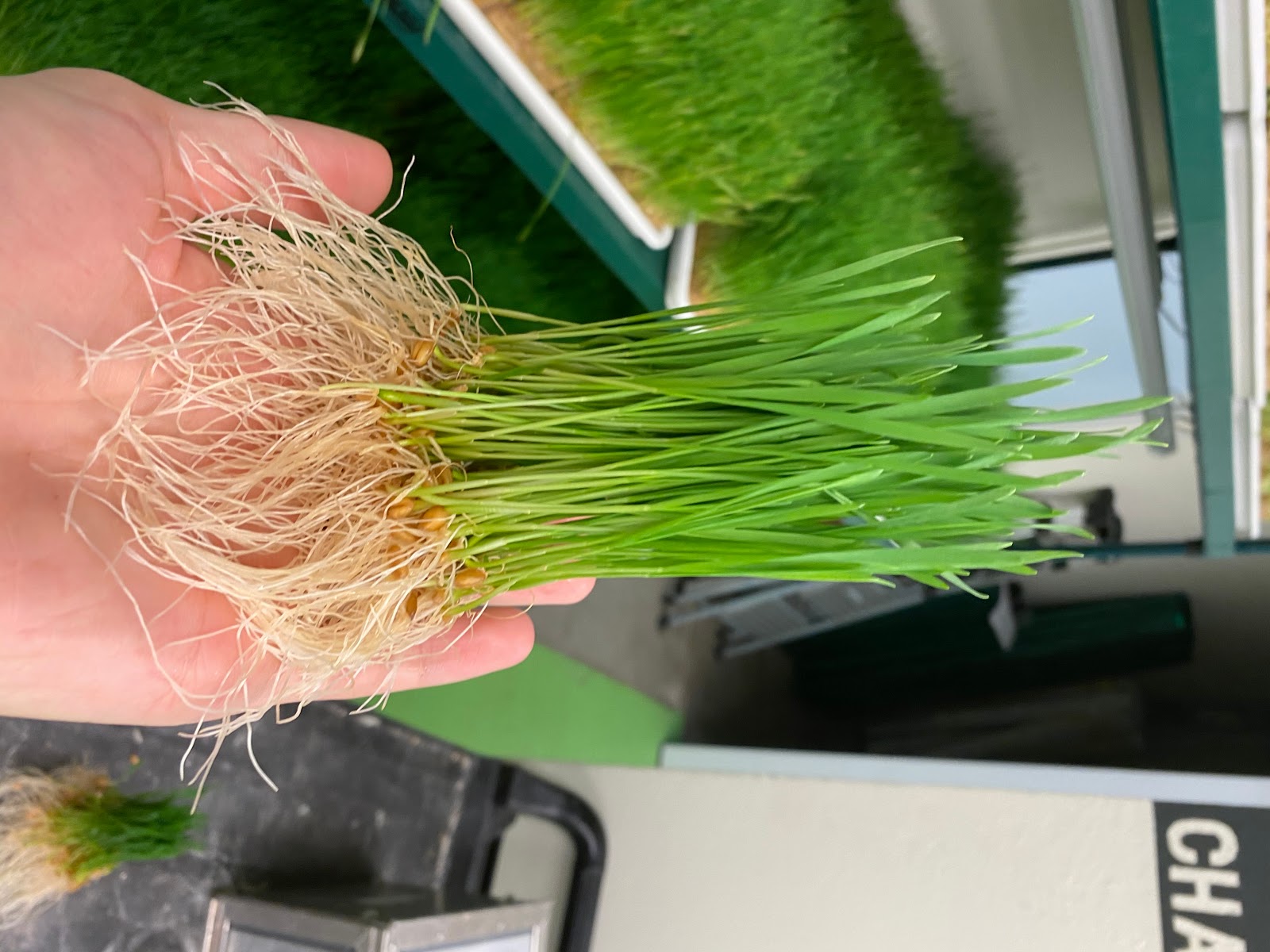


*Figure 1:* Visual representation of 3 week old wheat patty.

*Equation 1.*

The following equation was used for determining the dry mass for plants used in the experiments.

$$Y=0.0803x-0.0376$$

*Table 1:*

Regression stats for above equation.

|  | **Slope** | **Intercept** |
| --- | --- | --- |
| slope, intercept | 0.08033270975 | -0.03757617374 |
| SE slope, SE int | 0.01245219646 | 0.685466472 |
| R^2 | 0.7909505185 | 0.6180168776 |
| F stat w/ df | 41.61912118 | 11 |
| Regression SS, Resisdual SS | 15.89620945 | 4.201393471 |
